# Peripheral Neuromodulation: Type 2 Complex Regional Pain Syndrome Secondary to an Excisional Biopsy

**DOI:** 10.7759/cureus.96242

**Published:** 2025-11-06

**Authors:** João Francisco Gouveia, Marta A Caldeira, Duarte Correia

**Affiliations:** 1 Internal Medicine, Hospital Central do Funchal, Funchal, PRT; 2 Pain Management, Hospital Central do Funchal, Funchal, PRT; 3 Pain Medicine, Hospital Central do Funchal, Funchal, PRT

**Keywords:** complex regional pain syndrome, excisional biopsy, ganglionar tuberculosis, neuromodulation, peripheral nerve stimulation

## Abstract

Complex regional pain syndrome (CRPS) is a multifactorial and often refractory condition that involves hyperalgesia and allodynia of the extremities. Current treatments are mostly limited and short-lived; thus, different modalities of interventional pain control have come up to play a significant role in the management of CRPS.

The aim of this article is to present a single case from a Multidisciplinary Pain Management Center, regarding a wireless peripheral nerve stimulation (WPNS) treatment for a type II CRPS.

The European Organization for the Research and Treatment of Cancer Quality of Life Questionnaire (EORTC QLQ-C30) questionnaire was used as tool during an 18th month period follow-up.

A 43-year-old healthy woman, diagnosed with ganglionar tuberculosis and a refractory type II CRPS in the right upper extremity, following an excisional biopsy. She underwent placement of a permanent electrode in the right ulnar nerve. During 18 months, a progressive reduction of pain medication and an improvement in her quality of life associated with a remarkable relief in pain, allodynia, and temperature impairment was achieved.

Given its high acceptance, safety, and potential to influence neuronal plasticity, WPNS thus shows to be a promising and favorable tool in the multidisciplinary approach of CRPS.

## Introduction

Complex regional pain syndrome (CRPS) is a progressive array of painful conditions that includes a broad spectrum of sensory, autonomic, and motor features and often affects the upper and/or lower limbs [1-4]. Its prevalence is approximately 5.4-26.2 cases per 100,000 people, and data describe that being female, having an upper extremity injury, and suffering high-energy trauma are some risk factors that increase the risk of developing this disorder [1-7].

Regarding its pathophysiology, some contributors seem to be in the genesis of this syndrome, such as sensitization of the central and peripheral nervous systems, inflammation, possible genetic factors, sympatho-afferent coupling, autoimmunity, and mental health factors. [7].

There is no key test for CRPS yet, despite its many diagnostic criteria. The Budapest criteria, which are currently the accepted diagnostic criteria for CRPS, characterize CRPS as regional pain disproportionate in time and severity to the inciting event, which is usually traumatic. Moreover, CRPS often involves sensory and motor impairment, as well as autonomic dysregulation, that usually misses a clear dermatomal or peripheral nerve distribution pattern [1,8]. This condition can be further divided into two types (type I or II), based on the presence or absence of a nerve lesion, which characterizes the nature of chronic pain, in nociceptive or neuropathic, respectively. A third type (not otherwise specified) has also been proposed for those patients partially meeting criteria for either type I or II and lacking a better explanation for their symptoms and signs [3].

Many emerging treatments, such as physical therapy, medications (anticonvulsants, antidepressants, among others), peripheral nerve stimulation (PNS), transcutaneous electrical nerve stimulation (TENS), and sympathetic blocks, can be considered as a part of individualized, patient-centered care of this syndrome. In the foundations of some of these treatments, it is the gate control theory of pain, which implicates the inhibition of ascending noxious impulses through nonpainful stimulation of large-diameter sensory fibers, thus modulating the perception of pain [9,10]. This mechanism, underlying the analgesic effects of PNS, has been shown to be effective in the treatment of several painful conditions, including postherpetic neuralgia, posttraumatic neuropathy, cranial neuralgias, various headache disorders, and CRPS [11].

This article was previously posted to the Authorea preprint server on August 29, 2023.

## Case presentation

**Figure 1:**
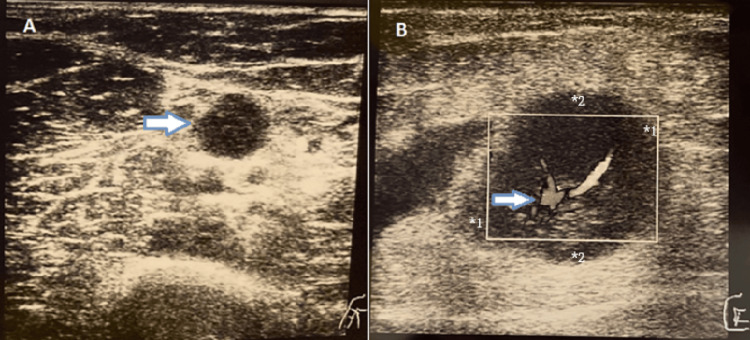
Right elbow ultrasound. Elbow ultrasound showed (A) an adenomegaly (arrow) with diffuse hyperechogenicity of the tissues and (B) an intensely vascularized adenomegaly (arrow) - diameter 1: 22.2 mm, diameter 2: 18.4 mm.

A 43-year-old healthy woman, whose father was diagnosed with pulmonary tuberculosis 20 years ago, presented with pain, swelling, and redness in the right elbow, associated with nodular purpuric lesions in the lower limbs. Her physical examination showed a painful, hard, and mobile adenomegaly at the level of the proximal region of the medial epicondyle of the right arm, and her right elbow ultrasound revealed an adenomegaly (Figure 1A), intensely vascularized (Figure 1B), with diffuse hyperechogenicity of the tissues (Figures 1A-1C).

**Figure 2:**
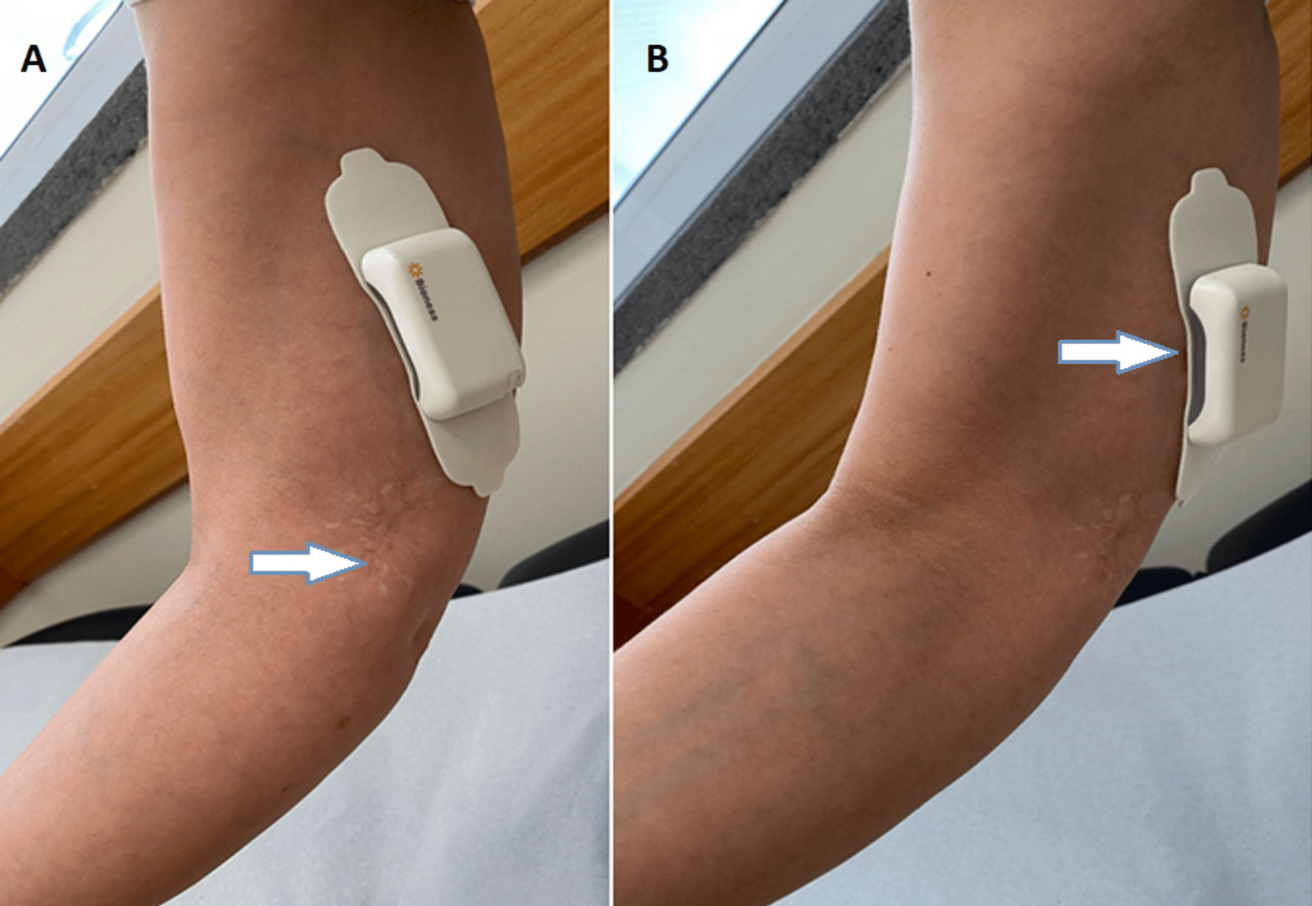
Excisional lymph node biopsy scar and peripheral nerve stimulator implanted in the right ulnar nerve. (A) Excisional lymph node biopsy scar (arrow) at the level of the proximal region of the medial epicondyle of the right arm. (B) Peripheral nerve stimulator (arrow) implanted in the right ulnar nerve.

Three months after lymph node biopsy, she clinically presented with a board of dysesthesia and allodynia of the right upper limb, essentially covering the territory of the medial antebrachial cutaneous nerve, with a positive Tinel sign above the biopsy scar (Figure 2A). At that time, therapy with gabapentin was started with 300 mg/day and progressively increased up to 1200 mg/day (300 mg/day dosage increase, every three weeks). Additionally, treatment with duloxetine was also started at 30 mg/day and increased up to 60 mg/day. One month after initiation of therapy, she was referred for a Pain Medicine consultation due to lack of pain improvement. Following the Pain Medicine consultation, an electromyography was asked and performed, which showed findings compatible with neuropraxia of the right ulnar nerve, in the subacute/chronic phase. A type 2 CRPS was assumed, and thereafter her medical treatment was adjusted. Other therapeutic options, such as tapentadol, 5% lidocaine patch, and 8% capsaicin, were tested, one month after the Pain Medicine consultation, but showed little efficacy. Three months after the beginning of the conventional treatment (Figure 2B), an easily implantable peripheral stimulator (StimRouter®, Bioness, Inc., Santa Clarita, CA) was placed at the level of the right ulnar nerve through a procedure under local anesthesia, due to sustained pain in the nerve distribution even after ulnar nerve block.

After these therapeutic adjustments had been carried out, there was a significant improvement in the symptoms. Four months after the peripheral stimulator was placed, the reduction of gabapentin dosage was then initiated, from 1200 mg/day (maximal dose) to 300 mg/day (300 mg/day reduction dosage, every three weeks). During an 18th month follow-up, with a high frequency (HF) stimulation (140 kHz/100 μs, 5.0 mA), it was documented a progressive reduction of pain medication, satisfactory pain control and subsequent improvement in her quality of life, according to the results of European Organization for the Research and Treatment of Cancer Quality of Life Questionnaire (EORTC QLQ-C30) questionnaire (included in the Appendices), completed by the patient previously and after the peripheral nerve stimulator implantation (Table 1).

**Table 1:** Results of questionnaire (EORTC QLQ-C30), completed by the patient previously and after the peripheral nerve stimulator implantation. Source: Questionnaire available via: http://qol.eortc.org.

Time (following peripheral stimulator implantation)	Questionnaire				
	EORTC QLQ-C30				
	PF2 (Physical functioning)	EF (Emotional functioning)	CF (Cognitive functioning)	PA (Pain)	QL2 (Global health status)
Zero month	86.7	67	83	100	50
15 months	93	100	100	50	91.7
18 months	100	100	100	50	100

## Discussion

CRPS is not a fully understood chronic pain condition, and its treatment options are still uncertain [6,8-11]. The disease course is variable, ranging from self-limiting to chronic, and can lead to progressively painful sensory and vascular changes, edema, limb weakness, and trophic disturbances, all of which substantially erode healthy living [12]. An incomplete understanding of the pathophysiology of the disease renders treatment challenging.

The most effective analgesia might be achieved by incorporating a variety of standard therapies with different modes of action. Introducing less conventional approaches may also be helpful when traditional treatments fail to provide sufficient improvement [12]. The current therapeutic options are mostly limited and short-lived and involve a multimodal approach of psychiatric and physical therapy, medical management, and interventional procedures [12-14]. The choice of which modality is more suitable will generally be determined by factors such as: pain localized to a specific nerve territory or pain that is felt particularly distal in an extremity [1]. Among the interventional procedures is the PNS [13,15]. Its application is recommended when a patient’s symptoms are refractory to conventional interventions such as physical therapy, medications, TENS, and sympathetic blocks [7,8,15]. 

This report of a refractory case of type II CRPS, caused by an excisional biopsy and ultimately managed with a peripheral nerve stimulator, may stem from the fact that the underlying pathophysiology in CRPS may not be uniform across all patients, and may even change within the same patient at various stages in their disease history. In fact, overall treatment failure can be high, and sustained long-lasting relief may be difficult to achieve in many patients [1-3,5].

EORTC QLQ-C30 is an instrument designed to measure quality of life or to determine and quantify perceived health problems [16]. This questionnaire was used with the aim of measuring the subjective health status of the patient, as chronic pain is a multidimensional pathological state that distorts specific brain properties and patients' psychological and physical traits [17]. In this case, the procedure was tolerated well, both electrodes remained in place without any adverse events, and the WPNS of the ulnar nerve provided good symptomatic relief. In view of the very limited options currently available to manage CRPS, WPNS can be a promising therapeutic modality.

## Conclusions

Although improvement had been reported, in this case, following the PNS, further follow-up is required to evaluate if there will be a sustained, long-lasting pain relief, and further trials would be necessary to confirm these findings.

A supplementary effort is, arguably, needed to explain the subgroups of patients who would benefit from the currently available treatments, including neuromodulation. In either way, a multidisciplinary approach is still the most recommended approach to date for managing CRPS patients.

## Figures and Tables

**Figure 3:**
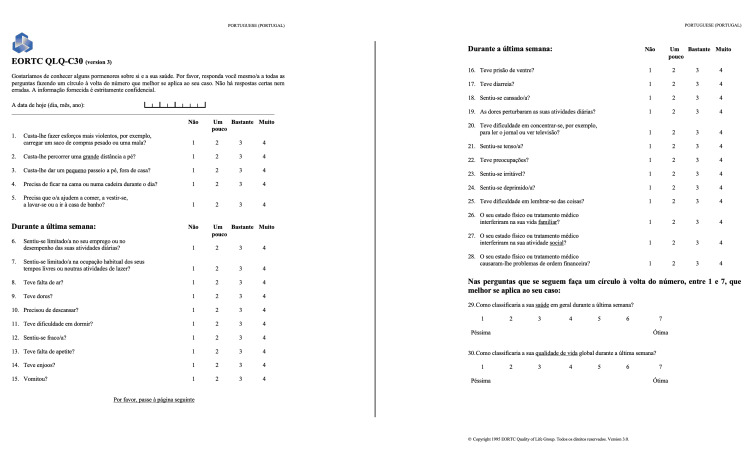
EORTC QLQ-C30: European Organization for the Research and Treatment of Cancer Quality of Life Questionnaire Source: Questionnaire available via: http://qol.eortc.org.
